# Supplementary material for: Monsoon weather and early childhood health in India
Source: PLoS One. 2020 Apr 10;15(4):e0231479. doi: 10.1371/journal.pone.0231479 (PMC7147999; doi:10.1371/journal.pone.0231479)
Supplement: S1 Appendix — (DOCX) [file pone.0231479.s001.docx]

# **S1 Appendix. Data**

**S1 Table. Descriptive statistics of individual, maternal and household control variables used in the regression**

| Variable | Unit | Mean  (Proportion) | Std. Dev. |
| --- | --- | --- | --- |
| Individual characteristics |  |  |  |
| Sex |  |  |  |
| Female* | 0/1 | 0.48 |  |
| Male | 0/1 | 0.52 |  |
| Birth order | number | 2.28 | 1.49 |
| Twin |  |  |  |
| No* | 0/1 | 0.98 |  |
| Yes | 0/1 | 0.02 |  |
| Low birth weight |  | 0.12 |  |
|  |  |  |  |
| Maternal characteristics |  |  |  |
| Mother’s age | years | 25.15 | 4.97 |
| Mother’s height | cm | 1516.75 | 60.90 |
| Exposed to mass media |  |  |  |
| No* | 0/1 | 0.37 |  |
| Yes | 0/1 | 0.63 |  |
| Education |  |  |  |
| No^*^ | 0/1 | 0.31 |  |
| Primary | 0/1 | 0.15 |  |
| Secondary | 0/1 | 0.45 |  |
| Higher | 0/1 | 0.09 |  |
|  |  |  |  |
| Household characteristics |  |  |  |
| Wealth quintile |  |  |  |
| 1^st^ (bottom)* | 0/1 | 0.27 |  |
| 2^nd^ | 0/1 | 0.24 |  |
| 3^rd^ | 0/1 | 0.20 |  |
| 4^st^ | 0/1 | 0.16 |  |
| 5^th^ (top) | 0/1 | 0.13 |  |
| Sanitation facility |  |  |  |
| Unimproved* | 0/1 | 0.58 |  |
| Improved | 0/1 | 0.42 |  |
| Household head |  |  |  |
| Male* | 0/1 | 0.88 |  |
| Female | 0/1 | 0.12 |  |
| Number of under-5 children | number | 1.72 | 0.95 |
| Caste |  |  |  |
| Other* | 0/1 | 0.22 |  |
| Scheduled caste | 0/1 | 0.19 |  |
| Scheduled tribe | 0/1 | 0.20 |  |
| Other backward caste | 0/1 | 0.39 |  |
| Religion |  |  |  |
| Hindu* | 0/1 | 0.73 |  |
| Muslim | 0/1 | 0.16 |  |
| Christian | 0/1 | 0.08 |  |
| Other | 0/1 | 0.04 |  |

Notes: *Used as a reference category in the regression.
