# Supplementary material for: Monsoon weather and early childhood health in India
Source: PLoS One. 2020 Apr 10;15(4):e0231479. doi: 10.1371/journal.pone.0231479 (PMC7147999; doi:10.1371/journal.pone.0231479)
Supplement: S2 Appendix — (DOCX) [file pone.0231479.s002.docx]

**S2 Appendix. Results tables**

**S2 Table. Effects of monsoon season climate during in-utero on undernutrition, children aged 0-5**

|  | HAZ | stunted  (HAZ<-2) | severely stunted  (HAZ<-3) | HAZ | stunted  (HAZ<-2) | severely stunted  (HAZ<-3) |
| --- | --- | --- | --- | --- | --- | --- |
|  | OLS Coef. | Odds Ratio | Odds Ratio | OLS Coef. | Odds Ratio | Odds Ratio |
| SPEI in utero | -0.034** | 1.047** | 1.033 |  |  |  |
|  | [-0.057, -0.011] | [1.016, 1.080] | [0.993, 1.075] |  |  |  |
| Drought in utero (SPEI≤-1.5) |  |  |  | 0.030+ | 0.954* | 0.98 |
|  |  |  |  | [-0.005, 0.065] | [0.911, 0.998] | [0.925, 1.039] |
| Flood in utero (SPEI≥1.5) |  |  |  | -0.027+ | 1.039* | 1.029 |
|  |  |  |  | [-0.054, 0.001] | [1.001, 1.078] | [0.980, 1.080] |
| Child is male | -0.117*** | 1.143*** | 1.272*** | -0.117*** | 1.143*** | 1.272*** |
|  | [-0.155, -0.080] | [1.087, 1.201] | [1.192, 1.357] | [-0.155, -0.079] | [1.087, 1.201] | [1.192, 1.357] |
| Birth order | -0.064*** | 1.080*** | 1.093*** | -0.064*** | 1.079*** | 1.093*** |
|  | [-0.076, -0.052] | [1.062, 1.097] | [1.072, 1.114] | [-0.076, -0.051] | [1.062, 1.097] | [1.072, 1.114] |
| Child is male: Birth order | 0.014+ | 0.982* | 0.960*** | 0.013+ | 0.982* | 0.960*** |
|  | [-0.001, 0.028] | [0.964, 1.000] | [0.940, 0.981] | [-0.001, 0.028] | [0.964, 1.000] | [0.940, 0.981] |
| Child is twin | -0.302*** | 1.476*** | 1.572*** | -0.301*** | 1.475*** | 1.571*** |
|  | [-0.413, -0.191] | [1.286, 1.693] | [1.335, 1.851] | [-0.412, -0.190] | [1.285, 1.692] | [1.334, 1.849] |
| Mother's age | 0.017*** | 0.981*** | 0.984*** | 0.017*** | 0.981*** | 0.984*** |
|  | [0.014, 0.020] | [0.978, 0.985] | [0.979, 0.988] | [0.014, 0.020] | [0.978, 0.985] | [0.979, 0.988] |
| Mother's height | 0.045*** | 0.939*** | 0.943*** | 0.044*** | 0.939*** | 0.943*** |
|  | [0.042, 0.047] | [0.936, 0.942] | [0.939, 0.946] | [0.042, 0.047] | [0.936, 0.942] | [0.939, 0.946] |
| Exposed to mass media | 0.024 | 0.969+ | 0.943* | 0.024 | 0.969+ | 0.943* |
|  | [-0.005,0.053] | [0.936, 1.003] | [0.900, 0.987] | [-0.005, 0.053] | [0.936, 1.003] | [0.900, 0.987] |
| Primary education | 0.039* | 0.949* | 0.842*** | 0.039* | 0.949* | 0.842*** |
|  | [0.004, 0.074] | [0.909, 0.991] | [0.798, 0.888] | [0.004, 0.074] | [0.909, 0.991] | [0.798, 0.888] |
| Secondary education | 0.102*** | 0.840*** | 0.771*** | 0.102*** | 0.840*** | 0.771*** |
|  | [0.071, 0.134] | [0.807, 0.873] | [0.734, 0.809] | [0.071, 0.134] | [0.807, 0.873] | [0.734, 0.809] |
| Higher education | 0.241*** | 0.673*** | 0.674*** | 0.241*** | 0.673*** | 0.674*** |
|  | [0.193, 0.289] | [0.630, 0.719] | [0.612, 0.742] | [0.193, 0.289] | [0.630, 0.719] | [0.612, 0.742] |
| 2nd wealth quintile | 0.088*** | 0.882*** | 0.806*** | 0.088*** | 0.883*** | 0.806*** |
|  | [0.055, 0.122] | [0.846, 0.920] | [0.765, 0.849] | [0.055, 0.122] | [0.846, 0.921] | [0.765, 0.849] |
| 3rd wealth quintile | 0.216*** | 0.756*** | 0.641*** | 0.216*** | 0.756*** | 0.641*** |
|  | [0.173, 0.258] | [0.717, 0.797] | [0.599, 0.686] | [0.173, 0.259] | [0.717, 0.797] | [0.599, 0.686] |
| 4th wealth quintile | 0.340*** | 0.615*** | 0.550*** | 0.340*** | 0.615*** | 0.550*** |
|  | [0.292, 0.389] | [0.577, 0.654] | [0.504, 0.600] | [0.292, 0.389] | [0.577, 0.654] | [0.504, 0.600] |
| Top wealth quintile | 0.462*** | 0.518*** | 0.504*** | 0.462*** | 0.518*** | 0.504*** |
|  | [0.403, 0.520] | [0.479, 0.561] | [0.452, 0.562] | [0.403, 0.520] | [0.479, 0.561] | [0.452, 0.562] |
| Sanitation facility is improved | 0.041** | 0.957* | 0.943* | 0.041** | 0.957* | 0.943* |
|  | [0.011, 0.070] | [0.922, 0.995] | [0.896, 0.991] | [0.011, 0.070] | [0.921, 0.995] | [0.896, 0.991] |
| Household head is female | -0.038* | 1.027 | 1.004 | -0.038* | 1.027 | 1.004 |
|  | [-0.071, -0.005] | [0.985, 1.070] | [0.952, 1.058] | [-0.071, -0.005] | [0.985, 1.070] | [0.952, 1.057] |
| Number of under-5 children | -0.036*** | 1.050*** | 1.045*** | -0.036*** | 1.050*** | 1.045*** |
|  | [-0.049, -0.023] | [1.032, 1.069] | [1.024, 1.066] | [-0.049, -0.023] | [1.032, 1.069] | [1.024, 1.066] |
| Scheduled caste | -0.151*** | 1.189*** | 1.202*** | -0.151*** | 1.188*** | 1.202*** |
|  | [-0.191, -0.110] | [1.125, 1.256] | [1.124, 1.286] | [-0.191, -0.110] | [1.125, 1.255] | [1.124, 1.286] |
| Scheduled tribe | -0.108*** | 1.165*** | 1.160*** | -0.108*** | 1.165*** | 1.160*** |
|  | [-0.159, -0.057] | [1.093, 1.241] | [1.069, 1.258] | [-0.159, -0.057] | [1.093, 1.241] | [1.069, 1.258] |
| Other backward caste | -0.073*** | 1.091*** | 1.085** | -0.073*** | 1.091*** | 1.085** |
|  | [-0.106, -0.039] | [1.045, 1.140] | [1.026, 1.147] | [-0.106, -0.039] | [1.045, 1.140] | [1.026, 1.147] |
| Muslim religion | -0.069*** | 1.102*** | 1.149*** | -0.069*** | 1.103*** | 1.150*** |
|  | [-0.108, -0.031] | [1.046, 1.162] | [1.080, 1.222] | [-0.108, -0.031] | [1.046, 1.162] | [1.081, 1.223] |
| Christian religion | 0.012 | 0.993 | 1.045 | 0.012 | 0.994 | 1.046 |
|  | [-0.067, 0.092] | [0.890, 1.108] | [0.898, 1.217] | [-0.067, 0.092] | [0.890, 1.109] | [0.898, 1.217] |
| Other religion | 0.087* | 0.954 | 0.983 | 0.087* | 0.954 | 0.983 |
|  | [0.012, 0.162] | [0.871, 1.044] | [0.862, 1.120] | [0.011, 0.162] | [0.872, 1.044] | [0.863, 1.120] |
| Obs. | 110,335 | 110,319 | 110,179 | 110,335 | 110,319 | 110,179 |
| (Pseudo) R^2^ | 0.151 | 0.096 | 0.088 | 0.151 | 0.096 | 0.088 |

+<0.1, * <0.05, ** <0.01, *** <0.001. Notes: 95% CIs provided in parenthesis. Age splines, month of birth, year of birth, and district fixed effects are included but not displayed. Clustering at the district level.

**S3 Table. Effects of monsoon season climate during infancy on undernutrition, children aged 0-5**

|  | HAZ | stunted  (HAZ<-2) | severely stunted  (HAZ<-3) | HAZ | stunted  (HAZ<-2) | severely stunted  (HAZ<-3) |
| --- | --- | --- | --- | --- | --- | --- |
|  | OLS Coef. | Odds Ratio | Odds Ratio | OLS Coef. | Odds Ratio | Odds Ratio |
| SPEI in infancy | -0.01 | 1.037** | 1.065*** |  |  |  |
|  | [-0.031, 0.010] | [1.011, 1.063] | [1.034, 1.096] |  |  |  |
| Drought in infancy (SPEI≤-1.5) |  |  |  | 0.017 | 0.966* | 0.945** |
|  |  |  |  | [-0.006, 0.040] | [0.935, 0.997] | [0.908, 0.984] |
| Flood in infancy (SPEI≥1.5) |  |  |  | -0.002 | 1.040** | 1.042* |
|  |  |  |  | [-0.023, 0.019] | [1.011, 1.070] | [1.006, 1.080] |
| Child is male | -0.109*** | 1.151*** | 1.229*** | -0.109*** | 1.151*** | 1.229*** |
|  | [-0.135, -0.082] | [1.108, 1.195] | [1.174, 1.288] | [-0.135, -0.082] | [1.108, 1.195] | [1.174, 1.287] |
| Birth order | -0.071*** | 1.098*** | 1.103*** | -0.071*** | 1.098*** | 1.103*** |
|  | [-0.080, -0.062] | [1.085, 1.111] | [1.087, 1.119] | [-0.080, -0.062] | [1.085, 1.111] | [1.087, 1.119] |
| Child is male: Birth order | 0.016** | 0.974*** | 0.970*** | 0.016** | 0.974*** | 0.970*** |
|  | [0.006, 0.026] | [0.961, 0.987] | [0.955, 0.984] | [0.006, 0.026] | [0.961, 0.987] | [0.955, 0.984] |
| Child is twin | -0.283*** | 1.430*** | 1.529*** | -0.283*** | 1.429*** | 1.528*** |
|  | [-0.366, -0.199] | [1.280, 1.597] | [1.346, 1.736] | [-0.366, -0.199] | [1.279, 1.596] | [1.345, 1.736] |
| Mother's age | 0.017*** | 0.981*** | 0.982*** | 0.017*** | 0.981*** | 0.982*** |
|  | [0.014, 0.019] | [0.978, 0.983] | [0.978, 0.986] | [0.014, 0.019] | [0.978, 0.983] | [0.978, 0.986] |
| Mother's height | 0.044*** | 0.937*** | 0.942*** | 0.044*** | 0.937*** | 0.942*** |
|  | [0.042, 0.046] | [0.934, 0.940] | [0.939, 0.945] | [0.042, 0.046] | [0.934, 0.940] | [0.939, 0.945] |
| Exposed to mass media | 0.047*** | 0.946*** | 0.900*** | 0.047*** | 0.946*** | 0.900*** |
|  | [0.024, 0.069] | [0.919, 0.974] | [0.870, 0.932] | [0.024, 0.069] | [0.919, 0.974] | [0.870, 0.932] |
| Primary education | 0.038** | 0.934*** | 0.863*** | 0.038** | 0.934*** | 0.863*** |
|  | [0.013, 0.063] | [0.905, 0.964] | [0.829, 0.897] | [0.013, 0.063] | [0.905, 0.964] | [0.829, 0.897] |
| Secondary education | 0.126*** | 0.817*** | 0.749*** | 0.126*** | 0.817*** | 0.749*** |
|  | [0.103, 0.149] | [0.792, 0.842] | [0.720, 0.778] | [0.103, 0.149] | [0.792, 0.842] | [0.720, 0.778] |
| Higher education | 0.256*** | 0.661*** | 0.622*** | 0.256*** | 0.661*** | 0.623*** |
|  | [0.218, 0.293] | [0.627, 0.697] | [0.577, 0.671] | [0.218, 0.293] | [0.627, 0.697] | [0.577, 0.671] |
| 2nd wealth quintile | 0.101*** | 0.870*** | 0.815*** | 0.101*** | 0.870*** | 0.815*** |
|  | [0.075, 0.126] | [0.842, 0.899] | [0.782, 0.849] | [0.075, 0.126] | [0.842, 0.899] | [0.782, 0.848] |
| 3rd wealth quintile | 0.216*** | 0.742*** | 0.656*** | 0.217*** | 0.741*** | 0.655*** |
|  | [0.183, 0.250] | [0.712, 0.773] | [0.621, 0.692] | [0.183, 0.250] | [0.711, 0.773] | [0.621, 0.692] |
| 4th wealth quintile | 0.339*** | 0.604*** | 0.547*** | 0.339*** | 0.604*** | 0.547*** |
|  | [0.300, 0.378] | [0.574, 0.635] | [0.509, 0.588] | [0.300, 0.378] | [0.574, 0.635] | [0.509, 0.588] |
| Top wealth quintile | 0.488*** | 0.497*** | 0.491*** | 0.489*** | 0.497*** | 0.491*** |
|  | [0.440, 0.537] | [0.465, 0.530] | [0.449, 0.536] | [0.440, 0.537] | [0.465, 0.530] | [0.449, 0.536] |
| Sanitation facility is improved | 0.053*** | 0.946*** | 0.941** | 0.053*** | 0.946*** | 0.942** |
|  | [0.031, 0.075] | [0.918, 0.974] | [0.905, 0.980] | [0.031, 0.075] | [0.918, 0.975] | [0.905, 0.980] |
| Household head is female | -0.014 | 1.013 | 0.994 | -0.014 | 1.014 | 0.994 |
|  | [-0.038, 0.010] | [0.981, 1.047] | [0.954, 1.036] | [-0.038, 0.010] | [0.981, 1.047] | [0.954, 1.036] |
| Number of under-5 children | -0.059*** | 1.068*** | 1.072*** | -0.059*** | 1.068*** | 1.072*** |
|  | [-0.069, -0.048] | [1.053, 1.083] | [1.056, 1.089] | [-0.069, -0.048] | [1.053, 1.083] | [1.056, 1.089] |
| Scheduled caste | -0.161*** | 1.233*** | 1.216*** | -0.161*** | 1.233*** | 1.216*** |
|  | [-0.192, -0.130] | [1.183, 1.285] | [1.151, 1.284] | [-0.192, -0.130] | [1.182, 1.285] | [1.151, 1.284] |
| Scheduled tribe | -0.105*** | 1.173*** | 1.189*** | -0.105*** | 1.173*** | 1.188*** |
|  | [-0.145, -0.065] | [1.114, 1.236] | [1.114, 1.269] | [-0.145, -0.065] | [1.113, 1.235] | [1.113, 1.269] |
| Other backward caste | -0.078*** | 1.115*** | 1.089*** | -0.078*** | 1.115*** | 1.089*** |
|  | [-0.105, -0.052] | [1.077, 1.156] | [1.040, 1.141] | [-0.105, -0.052] | [1.076, 1.155] | [1.040, 1.141] |
| Muslim religion | -0.081*** | 1.147*** | 1.165*** | -0.081*** | 1.146*** | 1.165*** |
|  | [-0.112, -0.050] | [1.100, 1.196] | [1.113, 1.220] | [-0.112, -0.050] | [1.099, 1.195] | [1.113, 1.219] |
| Christian religion | 0.032 | 0.966 | 0.944 | 0.032 | 0.966 | 0.945 |
|  | [-0.024, 0.088] | [0.893, 1.045] | [0.847, 1.054] | [-0.024, 0.088] | [0.894, 1.045] | [0.847, 1.054] |
| Other religion | 0.087** | 0.935+ | 0.957 | 0.087** | 0.936+ | 0.957 |
|  | [0.024, 0.150] | [0.868, 1.008] | [0.861, 1.063] | [0.024, 0.150] | [0.868, 1.008] | [0.861, 1.063] |
| Obs. | 188,732 | 188,712 | 188,709 | 188,732 | 188,712 | 188,709 |
| (Pseudo) R^2^ | 0.136 | 0.094 | 0.092 | 0.136 | 0.094 | 0.092 |

+<0.1, * <0.05, ** <0.01, *** <0.001. Notes: 95% CIs provided in parenthesis. Age splines, month of birth, year of birth, and district fixed effects are included but not displayed. Clustering at the district level.

**S4 Table. Effects of monsoon season climate prior to interview on undernutrition, children aged 0-5**

|  | WHZ | wasted  (WHZ<-2) | severely wasted  (WHZ <-3) | WHZ | wasted  (WHZ<-2) | severely wasted  (WHZ<-3) |
| --- | --- | --- | --- | --- | --- | --- |
|  | OLS Coef. | Odds Ratio | Odds Ratio | OLS Coef. | Odds Ratio | Odds Ratio |
| SPEI latest monsoon season | -0.023 | 1.036 | 0.928 |  |  |  |
|  | [-0.082,0.036] | [0.942,1.140] | [0.802,1.075] |  |  |  |
| Drought latest monsoon season (SPEI≤-1.5) |  |  |  | 0.059* | 0.889*** | 0.918 |
|  |  |  |  | [0.013,0.105] | [0.831,0.950] | [0.822,1.023] |
| Flood latest monsoon season (SPEI≥1.5) |  |  |  | 0.062* | 0.910+ | 0.837* |
|  |  |  |  | [0.009,0.114] | [0.827,1.002] | [0.719,0.975] |
| Child is male | -0.058*** | 1.155*** | 1.210*** | -0.058*** | 1.155*** | 1.210*** |
|  | [-0.080,-0.036] | [1.108,1.204] | [1.136,1.289] | [-0.080,-0.036] | [1.108,1.204] | [1.136,1.289] |
| Birth order | -0.016*** | 1.012 | 1.025* | -0.016*** | 1.012 | 1.025* |
|  | [-0.024,-0.009] | [0.997,1.026] | [1.002,1.048] | [-0.024,-0.009] | [0.997,1.026] | [1.002,1.048] |
| Child is male: Birth order | 0.003 | 0.996 | 0.994 | 0.003 | 0.996 | 0.994 |
|  | [-0.005,0.011] | [0.981,1.012] | [0.970,1.018] | [-0.005,0.011] | [0.980,1.012] | [0.970,1.018] |
| Child is twin | -0.170*** | 1.298*** | 1.163+ | -0.169*** | 1.297*** | 1.163+ |
|  | [-0.229,-0.111] | [1.167,1.443] | [0.978,1.383] | [-0.228,-0.110] | [1.166,1.442] | [0.978,1.383] |
| Mother's age | 0.004*** | 0.998 | 0.995+ | 0.004*** | 0.998 | 0.995+ |
|  | [0.002,0.005] | [0.994,1.001] | [0.990,1.000] | [0.002,0.005] | [0.994,1.001] | [0.990,1.000] |
| Mother's height | 0.008*** | 0.991*** | 0.994** | 0.008*** | 0.991*** | 0.994** |
|  | [0.006,0.009] | [0.989,0.994] | [0.990,0.997] | [0.006,0.009] | [0.989,0.994] | [0.990,0.997] |
| Exposed to mass media | 0.004 | 0.99 | 0.955+ | 0.004 | 0.99 | 0.955+ |
|  | [-0.015,0.023] | [0.956,1.024] | [0.906,1.007] | [-0.015,0.023] | [0.956,1.025] | [0.906,1.007] |
| Primary education | 0.029** | 0.922*** | 0.919** | 0.029** | 0.922*** | 0.919** |
|  | [0.008,0.051] | [0.887,0.959] | [0.864,0.977] | [0.008,0.051] | [0.887,0.959] | [0.865,0.978] |
| Secondary education | 0.041*** | 0.915*** | 0.889*** | 0.040*** | 0.915*** | 0.890*** |
|  | [0.022,0.059] | [0.882,0.948] | [0.842,0.939] | [0.022,0.059] | [0.883,0.948] | [0.842,0.940] |
| Higher education | 0.149*** | 0.836*** | 0.875** | 0.149*** | 0.836*** | 0.874** |
|  | [0.117,0.181] | [0.784,0.891] | [0.794,0.964] | [0.117,0.181] | [0.784,0.890] | [0.793,0.963] |
| 2nd wealth quintile | 0.045*** | 0.888*** | 0.858*** | 0.045*** | 0.887*** | 0.858*** |
|  | [0.025,0.066] | [0.852,0.925] | [0.810,0.910] | [0.025,0.066] | [0.851,0.925] | [0.809,0.909] |
| 3rd wealth quintile | 0.094*** | 0.826*** | 0.801*** | 0.094*** | 0.826*** | 0.801*** |
|  | [0.068,0.120] | [0.785,0.869] | [0.742,0.865] | [0.068,0.120] | [0.786,0.869] | [0.742,0.865] |
| 4th wealth quintile | 0.152*** | 0.772*** | 0.747*** | 0.152*** | 0.772*** | 0.746*** |
|  | [0.119,0.184] | [0.728,0.819] | [0.688,0.810] | [0.120,0.184] | [0.728,0.818] | [0.687,0.809] |
| Top wealth quintile | 0.246*** | 0.712*** | 0.728*** | 0.246*** | 0.712*** | 0.728*** |
|  | [0.206,0.287] | [0.662,0.765] | [0.654,0.810] | [0.206,0.287] | [0.662,0.765] | [0.654,0.810] |
| Sanitation facility is improved | 0.027** | 0.967* | 1.006 | 0.027** | 0.967* | 1.007 |
|  | [0.008,0.045] | [0.935,1.000] | [0.954,1.061] | [0.008,0.045] | [0.935,1.000] | [0.955,1.062] |
| Household head is female | 0.005 | 0.975 | 1.024 | 0.005 | 0.976 | 1.025 |
|  | [-0.016,0.026] | [0.939,1.013] | [0.965,1.087] | [-0.016,0.026] | [0.940,1.014] | [0.965,1.088] |
| Number of under-5 children | 0.017*** | 0.951*** | 0.920*** | 0.017*** | 0.951*** | 0.921*** |
|  | [0.008,0.025] | [0.935,0.967] | [0.896,0.945] | [0.008,0.025] | [0.936,0.967] | [0.896,0.945] |
| Scheduled caste | -0.074*** | 1.070** | 1.046 | -0.075*** | 1.071** | 1.047 |
|  | [-0.102,-0.046] | [1.018,1.125] | [0.969,1.129] | [-0.103,-0.047] | [1.019,1.126] | [0.970,1.131] |
| Scheduled tribe | -0.091*** | 1.166*** | 1.183*** | -0.092*** | 1.168*** | 1.186*** |
|  | [-0.130,-0.052] | [1.094,1.241] | [1.082,1.294] | [-0.131,-0.053] | [1.097,1.244] | [1.084,1.297] |
| Other backward caste | -0.051*** | 1.052* | 1.068+ | -0.051*** | 1.053* | 1.069+ |
|  | [-0.074,-0.027] | [1.008,1.098] | [0.997,1.144] | [-0.074,-0.028] | [1.008,1.099] | [0.998,1.145] |
| Muslim religion | -0.032* | 1.051* | 1.032 | -0.032* | 1.049* | 1.031 |
|  | [-0.059,-0.005] | [1.001,1.102] | [0.957,1.113] | [-0.059,-0.005] | [1.000,1.101] | [0.956,1.112] |
| Christian religion | 0.018 | 1.026 | 0.967 | 0.018 | 1.026 | 0.967 |
|  | [-0.051,0.087] | [0.914,1.152] | [0.827,1.131] | [-0.050,0.087] | [0.914,1.152] | [0.827,1.131] |
| Other religion | -0.008 | 1.026 | 1.153* | -0.007 | 1.024 | 1.150* |
|  | [-0.061,0.044] | [0.936,1.124] | [1.018,1.306] | [-0.059,0.045] | [0.934,1.122] | [1.016,1.303] |
| Obs. | 188,532 | 188,529 | 188,375 | 188,532 | 188,529 | 188,375 |
| (Pseudo) R^2^ | 0.078 | 0.053 | 0.063 | 0.078 | 0.053 | 0.063 |

+<0.1, * <0.05, ** <0.01, *** <0.001. Notes: 95% CIs provided in parenthesis. Age splines, month of birth, year of birth, and district fixed effects are included but not displayed. Clustering at the district level.

**S5 Table. Effects of SPEI in the month of interview on the risk of diarrhoea, children aged 0-5**

|  | Diarrhoea |  | diarrhoea |  |
| --- | --- | --- | --- | --- |
|  | Odds Ratio |  | Odds Ratio |  |
| SPEI month of interview | 1.066* | [1.011,1.124] |  |  |
|  |  |  |  |  |
| SPEI month of interview: Winter season |  |  | 1.08 | [0.976,1.195] |
| SPEI month of interview: Summer season |  |  | 1.016 | [0.941,1.097] |
| SPEI month of interview: Monsoon season |  |  | 1.159** | [1.051,1.279] |
| SPEI month of interview: Post-monsoon season |  |  | 1.193 | [0.958,1.486] |
|  |  |  |  |  |
| Summer season |  |  | 1.046 | [0.907,1.207] |
| Monsoon season |  |  | 1.005 | [0.842,1.198] |
| Post-monsoon season |  |  | 0.918 | [0.705,1.195] |
|  |  |  |  |  |
| Child is male | 1.112*** | [1.057,1.169] | 1.110*** | [1.049,1.175] |
| Birth order | 1.040*** | [1.021,1.060] | 1.038*** | [1.017,1.059] |
| Child is male: Birth order | 0.989 | [0.972,1.007] | 0.989 | [0.969,1.009] |
| Child is twin | 0.889 | [0.761,1.039] | 0.883 | [0.744,1.048] |
| Mother's age | 0.986*** | [0.982,0.991] | 0.987*** | [0.982,0.992] |
| Mother's height | 1.002 | [0.999,1.004] | 1.001 | [0.999,1.004] |
| Exposed to mass media | 1.013 | [0.968,1.060] | 1.026 | [0.976,1.078] |
| Primary education | 1.182*** | [1.122,1.245] | 1.149*** | [1.086,1.216] |
| Secondary education | 1.117*** | [1.062,1.175] | 1.111*** | [1.050,1.174] |
| Higher education | 1.012 | [0.935,1.095] | 0.995 | [0.913,1.084] |
| 2nd wealth quintile | 0.98 | [0.931,1.032] | 0.97 | [0.917,1.026] |
| 3rd wealth quintile | 0.959 | [0.899,1.023] | 0.939+ | [0.876,1.006] |
| 4th wealth quintile | 0.947 | [0.876,1.023] | 0.912* | [0.839,0.991] |
| Top wealth quintile | 0.884** | [0.812,0.963] | 0.853*** | [0.778,0.935] |
| Sanitation facility is improved | 0.933** | [0.892,0.976] | 0.941* | [0.897,0.987] |
| Household head is female | 1.011 | [0.963,1.061] | 0.998 | [0.946,1.053] |
| Number of under-5 children | 0.883*** | [0.864,0.902] | 0.882*** | [0.862,0.902] |
| Scheduled caste | 1.029 | [0.971,1.091] | 1.017 | [0.955,1.083] |
| Scheduled tribe | 0.919* | [0.848,0.995] | 0.906* | [0.830,0.988] |
| Other backward caste | 1.009 | [0.958,1.063] | 1.012 | [0.956,1.072] |
| Muslim religion | 1.158*** | [1.094,1.227] | 1.159*** | [1.091,1.232] |
| Christian religion | 1.028 | [0.870,1.214] | 0.999 | [0.850,1.176] |
| Other religion | 1.166* | [1.007,1.350] | 1.134 | [0.962,1.338] |
| Obs. | 225,577 |  | 188,642 |  |
| Pseudo R^2^ | 0.086 |  | 0.086 |  |

+<0.1, * <0.05, ** <0.01, *** <0.001. Notes: 95% CIs provided in parenthesis. Age splines, month of birth, year of birth, and district fixed effects are included but not displayed. Clustering at the district level.

**S6 Table. Effects of monsoon season SPEI on undernutrition by type of sanitation facility, children aged 0-5**

|  | stunted | stunted | wasted |
| --- | --- | --- | --- |
|  | Odds ratio | Odds ratio | Odds ratio |
| SPEI in utero: Improved sanitation facility | 1.060*** |  |  |
|  | [1.024,1.097] |  |  |
| SPEI in utero: Unimproved sanitation facility | 1.022 |  |  |
|  | [0.975,1.071] |  |  |
| SPEI in infancy: Improved sanitation facility |  | 1.054*** |  |
|  |  | [1.024,1.085] |  |
| SPEI in infancy: Unimproved sanitation facility |  | 1.003 |  |
|  |  | [0.967,1.040] |  |
| SPEI latest monsoon season: Improved sanitation facility |  |  | 1.039 |
|  |  |  | [0.944,1.144] |
| SPEI latest monsoon season: Unimproved sanitation facility |  |  | 1.032 |
|  |  |  | [0.931,1.144] |
| Child is male | 1.143*** | 1.151*** | 1.155*** |
|  | [1.087,1.201] | [1.108,1.195] | [1.108,1.204] |
| Birth order | 1.080*** | 1.098*** | 1.012 |
|  | [1.062,1.097] | [1.085,1.111] | [0.997,1.026] |
| Child is male: Birth order | 0.982* | 0.974*** | 0.996 |
|  | [0.964,1.000] | [0.961,0.987] | [0.981,1.012] |
| Child is twin | 1.475*** | 1.429*** | 1.298*** |
|  | [1.286,1.692] | [1.280,1.596] | [1.167,1.443] |
| Mother's age | 0.981*** | 0.981*** | 0.998 |
|  | [0.978,0.985] | [0.978,0.983] | [0.994,1.001] |
| Mother's height | 0.939*** | 0.937*** | 0.991*** |
|  | [0.936,0.942] | [0.934,0.940] | [0.989,0.994] |
| Exposed to mass media | 0.969+ | 0.946*** | 0.99 |
|  | [0.936,1.003] | [0.919,0.974] | [0.956,1.024] |
| Primary education | 0.949* | 0.934*** | 0.922*** |
|  | [0.909,0.991] | [0.905,0.964] | [0.887,0.959] |
| Secondary education | 0.840*** | 0.817*** | 0.915*** |
|  | [0.807,0.874] | [0.792,0.842] | [0.882,0.948] |
| Higher education | 0.673*** | 0.660*** | 0.836*** |
|  | [0.630,0.719] | [0.626,0.696] | [0.784,0.890] |
| 2nd wealth quintile | 0.882*** | 0.869*** | 0.887*** |
|  | [0.846,0.920] | [0.841,0.898] | [0.851,0.925] |
| 3rd wealth quintile | 0.755*** | 0.741*** | 0.826*** |
|  | [0.717,0.796] | [0.711,0.773] | [0.785,0.869] |
| 4th wealth quintile | 0.615*** | 0.604*** | 0.772*** |
|  | [0.577,0.654] | [0.574,0.636] | [0.728,0.818] |
| Top wealth quintile | 0.518*** | 0.497*** | 0.712*** |
|  | [0.479,0.561] | [0.466,0.531] | [0.662,0.765] |
| Sanitation facility is improved | 0.960* | 0.948*** | 0.966* |
|  | [0.924,0.997] | [0.920,0.977] | [0.933,1.000] |
| Household head is female | 1.027 | 1.014 | 0.976 |
|  | [0.985,1.070] | [0.981,1.047] | [0.939,1.013] |
| Number of under-5 children | 1.050*** | 1.068*** | 0.951*** |
|  | [1.032,1.069] | [1.053,1.083] | [0.935,0.967] |
| Scheduled caste | 1.189*** | 1.233*** | 1.071** |
|  | [1.125,1.256] | [1.183,1.286] | [1.018,1.125] |
| Scheduled tribe | 1.164*** | 1.172*** | 1.165*** |
|  | [1.093,1.241] | [1.113,1.235] | [1.094,1.241] |
| Other backward caste | 1.091*** | 1.115*** | 1.052* |
|  | [1.045,1.140] | [1.076,1.155] | [1.008,1.098] |
| Muslim religion | 1.102*** | 1.146*** | 1.050* |
|  | [1.046,1.161] | [1.099,1.195] | [1.001,1.102] |
| Christian religion | 0.993 | 0.966 | 1.026 |
|  | [0.889,1.108] | [0.893,1.045] | [0.914,1.152] |
| Other religion | 0.953 | 0.935+ | 1.026 |
|  | [0.871,1.044] | [0.868,1.008] | [0.936,1.124] |
| Obs. | 110,319 | 188,712 | 188,529 |
| Pseudo R^2^ | 0.096 | 0.094 | 0.053 |

+<0.1, * <0.05, ** <0.01, *** <0.001. Notes: 95% CIs provided in parenthesis. Age splines, month of birth, year of birth, and district fixed effects are included but not displayed. Clustering at the district level.

**S7 Table. Effects of monsoon season SPEI during in-utero on the risk of stunting by age at measurement**

|  | Age > 1 | Age > 2 | Age > 3 | Age > 4 |
| --- | --- | --- | --- | --- |
|  | Odds Ratio | Odds Ratio | Odds Ratio | Odds Ratio |
| SPEI in utero | 1.011 | 0.998 | 0.989 | 1.031 |
|  | [0.979,1.043] | [0.964,1.034] | [0.950,1.029] | [0.918,1.157] |
| Child is male | 1.108*** | 1.031 | 0.964 | 0.856** |
|  | [1.049,1.170] | [0.967,1.098] | [0.894,1.040] | [0.770,0.952] |
| Birth order | 1.091*** | 1.096*** | 1.102*** | 1.087*** |
|  | [1.073,1.110] | [1.075,1.117] | [1.078,1.127] | [1.052,1.124] |
| Child is male: Birth order | 0.983+ | 0.986 | 0.99 | 1.007 |
|  | [0.964,1.003] | [0.965,1.008] | [0.965,1.015] | [0.971,1.045] |
| Child is twin | 1.324*** | 1.282** | 1.469*** | 1.582* |
|  | [1.133,1.546] | [1.065,1.544] | [1.171,1.842] | [1.112,2.250] |
| Mother's age | 0.980*** | 0.978*** | 0.976*** | 0.977*** |
|  | [0.976,0.984] | [0.973,0.982] | [0.970,0.982] | [0.969,0.985] |
| Mother's height | 0.935*** | 0.933*** | 0.931*** | 0.931*** |
|  | [0.932,0.939] | [0.929,0.937] | [0.927,0.936] | [0.925,0.938] |
| Exposed to mass media | 0.968+ | 0.961+ | 0.967 | 1.012 |
|  | [0.931,1.006] | [0.920,1.005] | [0.914,1.023] | [0.931,1.100] |
| Primary education | 0.959+ | 0.957 | 0.934* | 0.926 |
|  | [0.915,1.005] | [0.907,1.010] | [0.876,0.996] | [0.843,1.017] |
| Secondary education | 0.835*** | 0.836*** | 0.837*** | 0.812*** |
|  | [0.798,0.874] | [0.794,0.881] | [0.787,0.890] | [0.743,0.889] |
| Higher education | 0.643*** | 0.622*** | 0.640*** | 0.548*** |
|  | [0.598,0.692] | [0.568,0.682] | [0.570,0.718] | [0.463,0.647] |
| 2nd wealth quintile | 0.877*** | 0.867*** | 0.848*** | 0.826*** |
|  | [0.837,0.919] | [0.821,0.915] | [0.794,0.906] | [0.751,0.908] |
| 3rd wealth quintile | 0.751*** | 0.739*** | 0.730*** | 0.719*** |
|  | [0.709,0.795] | [0.691,0.790] | [0.674,0.791] | [0.644,0.804] |
| 4th wealth quintile | 0.604*** | 0.591*** | 0.587*** | 0.541*** |
|  | [0.564,0.648] | [0.546,0.640] | [0.533,0.646] | [0.471,0.620] |
| Top wealth quintile | 0.503*** | 0.484*** | 0.473*** | 0.441*** |
|  | [0.461,0.548] | [0.437,0.537] | [0.417,0.535] | [0.368,0.528] |
| Sanitation facility is improved | 0.946* | 0.942* | 0.937* | 0.958 |
|  | [0.907,0.987] | [0.897,0.989] | [0.885,0.993] | [0.878,1.045] |
| Household head is female | 1.023 | 1.032 | 1.019 | 1.077 |
|  | [0.977,1.070] | [0.978,1.088] | [0.954,1.090] | [0.974,1.190] |
| Number of under-5 children | 1.070*** | 1.059*** | 1.042** | 0.991 |
|  | [1.049,1.091] | [1.036,1.084] | [1.015,1.070] | [0.953,1.031] |
| Scheduled caste | 1.192*** | 1.181*** | 1.191*** | 1.148* |
|  | [1.124,1.264] | [1.107,1.261] | [1.104,1.286] | [1.029,1.279] |
| Scheduled tribe | 1.165*** | 1.166*** | 1.153** | 1.156* |
|  | [1.087,1.248] | [1.075,1.265] | [1.049,1.268] | [1.005,1.329] |
| Other backward caste | 1.099*** | 1.088** | 1.096** | 1.091+ |
|  | [1.047,1.153] | [1.031,1.147] | [1.025,1.171] | [0.991,1.202] |
| Muslim religion | 1.120*** | 1.131*** | 1.149*** | 1.127* |
|  | [1.059,1.185] | [1.063,1.204] | [1.066,1.238] | [1.013,1.254] |
| Christian religion | 1.009 | 1.007 | 1.039 | 1.097 |
|  | [0.895,1.137] | [0.879,1.154] | [0.880,1.227] | [0.866,1.389] |
| Other religion | 0.962 | 0.993 | 1.035 | 0.976 |
|  | [0.870,1.064] | [0.884,1.116] | [0.892,1.201] | [0.791,1.204] |
| Obs. | 88,783 | 66,565 | 44,623 | 21,667 |
| Pseudo R^2^ | 0.091 | 0.101 | 0.107 | 0.116 |

+<0.1, * <0.05, ** <0.01, *** <0.001. Notes: 95% CIs provided in parenthesis. Age splines, month of birth, year of birth, and district fixed effects are included but not displayed. Clustering at the district level.

**S8 Table. Effects of monsoon season SPEI during infancy on the risk of stunting by age at measurement**

|  | Age > 1 | Age > 2 | Age > 3 | Age > 4 |
| --- | --- | --- | --- | --- |
|  | Odds Ratio | Odds Ratio | Odds Ratio | Odds Ratio |
| SPEI in infancy | 1.034** | 1.029+ | 1.029 | 1.024 |
|  | [1.008,1.060] | [0.999,1.060] | [0.992,1.068] | [0.974,1.075] |
| Child is male | 1.128*** | 1.067** | 1.031 | 0.96 |
|  | [1.085,1.171] | [1.020,1.116] | [0.977,1.088] | [0.892,1.034] |
| Birth order | 1.100*** | 1.109*** | 1.117*** | 1.108*** |
|  | [1.087,1.113] | [1.094,1.124] | [1.099,1.135] | [1.082,1.134] |
| Child is male: Birth order | 0.978** | 0.979** | 0.973** | 0.982 |
|  | [0.965,0.991] | [0.964,0.994] | [0.955,0.991] | [0.958,1.007] |
| Child is twin | 1.370*** | 1.350*** | 1.471*** | 1.497*** |
|  | [1.225,1.533] | [1.186,1.537] | [1.255,1.723] | [1.177,1.904] |
| Mother's age | 0.980*** | 0.977*** | 0.977*** | 0.977*** |
|  | [0.977,0.983] | [0.974,0.981] | [0.973,0.981] | [0.971,0.982] |
| Mother's height | 0.936*** | 0.934*** | 0.933*** | 0.934*** |
|  | [0.934,0.939] | [0.931,0.937] | [0.930,0.936] | [0.930,0.938] |
| Exposed to mass media | 0.948*** | 0.942*** | 0.939** | 0.952+ |
|  | [0.920,0.976] | [0.911,0.974] | [0.901,0.980] | [0.899,1.007] |
| Primary education | 0.934*** | 0.929*** | 0.924*** | 0.933* |
|  | [0.905,0.964] | [0.895,0.964] | [0.883,0.967] | [0.876,0.995] |
| Secondary education | 0.815*** | 0.818*** | 0.820*** | 0.807*** |
|  | [0.790,0.841] | [0.789,0.847] | [0.786,0.855] | [0.758,0.859] |
| Higher education | 0.656*** | 0.637*** | 0.634*** | 0.600*** |
|  | [0.621,0.692] | [0.595,0.681] | [0.583,0.689] | [0.531,0.678] |
| 2nd wealth quintile | 0.868*** | 0.865*** | 0.864*** | 0.853*** |
|  | [0.839,0.898] | [0.831,0.900] | [0.823,0.907] | [0.798,0.911] |
| 3rd wealth quintile | 0.739*** | 0.735*** | 0.735*** | 0.724*** |
|  | [0.708,0.771] | [0.700,0.772] | [0.694,0.779] | [0.669,0.784] |
| 4th wealth quintile | 0.601*** | 0.591*** | 0.601*** | 0.584*** |
|  | [0.570,0.633] | [0.556,0.627] | [0.561,0.644] | [0.530,0.642] |
| Top wealth quintile | 0.491*** | 0.485*** | 0.493*** | 0.480*** |
|  | [0.459,0.526] | [0.448,0.525] | [0.452,0.538] | [0.424,0.544] |
| Sanitation facility is improved | 0.948*** | 0.943** | 0.926*** | 0.96 |
|  | [0.919,0.978] | [0.910,0.978] | [0.889,0.964] | [0.906,1.018] |
| Household head is female | 1.013 | 1.01 | 0.993 | 1.002 |
|  | [0.980,1.047] | [0.972,1.050] | [0.949,1.039] | [0.939,1.070] |
| Number of under-5 children | 1.071*** | 1.068*** | 1.050*** | 1.008 |
|  | [1.056,1.087] | [1.050,1.085] | [1.030,1.071] | [0.980,1.037] |
| Scheduled caste | 1.237*** | 1.229*** | 1.221*** | 1.206*** |
|  | [1.185,1.290] | [1.172,1.290] | [1.157,1.289] | [1.119,1.300] |
| Scheduled tribe | 1.174*** | 1.191*** | 1.160*** | 1.176*** |
|  | [1.114,1.238] | [1.125,1.262] | [1.088,1.237] | [1.073,1.290] |
| Other backward caste | 1.117*** | 1.122*** | 1.122*** | 1.113** |
|  | [1.077,1.158] | [1.076,1.169] | [1.067,1.180] | [1.037,1.194] |
| Muslim religion | 1.144*** | 1.154*** | 1.155*** | 1.122** |
|  | [1.096,1.194] | [1.101,1.210] | [1.094,1.221] | [1.042,1.208] |
| Christian religion | 0.958 | 0.966 | 0.97 | 0.925 |
|  | [0.884,1.039] | [0.879,1.061] | [0.870,1.083] | [0.788,1.085] |
| Other religion | 0.938 | 0.955 | 0.981 | 0.916 |
|  | [0.867,1.015] | [0.871,1.046] | [0.881,1.091] | [0.788,1.065] |
| Obs. | 177,273 | 133,894 | 90,126 | 44,127 |
| Pseudo R^2^ | 0.089 | 0.097 | 0.099 | 0.102 |

+<0.1, * <0.05, ** <0.01, *** <0.001. Notes: 95% CIs provided in parenthesis. Age splines, month of birth, year of birth, and district fixed effects are included but not displayed. Clustering at the district level.

**S9 Table. Effects of monsoon season SPEI on child health by climate zone, children aged 0-5**

|  | stunted |  | stunted |  | wasted |  | diarrhoea |  |
| --- | --- | --- | --- | --- | --- | --- | --- | --- |
|  | Odds Ratio |  | Odds Ratio |  | Odds Ratio |  | Odds Ratio |  |
| **SPEI in utero: Climate zone** |  |  |  |  |  |  |  |  |
| SPEI in utero: Tropical wet | 1.012 | [0.880,1.164] |  |  |  |  |  |  |
| SPEI in utero: Tropical wet & dry | 0.99 | [0.923,1.062] |  |  |  |  |  |  |
| SPEI in utero: Arid | 1.039 | [0.799,1.353] |  |  |  |  |  |  |
| SPEI in utero: Semi-arid | 0.996 | [0.911,1.088] |  |  |  |  |  |  |
| SPEI in utero: Humid sub-tropical | 1.077*** | [1.038,1.118] |  |  |  |  |  |  |
| SPEI in utero: Mountain | 0.891 | [0.660,1.204] |  |  |  |  |  |  |
|  |  |  |  |  |  |  |  |  |
| **SPEI in infancy: Climate zone** |  |  |  |  |  |  |  |  |
| SPEI in infancy: Tropical wet |  |  | 1.179** | [1.045,1.329] |  |  |  |  |
| SPEI in infancy: Tropical wet & dry |  |  | 1.002 | [0.955,1.052] |  |  |  |  |
| SPEI in infancy: Arid |  |  | 0.991 | [0.834,1.179] |  |  |  |  |
| SPEI in infancy: Semi-arid |  |  | 1.029 | [0.962,1.101] |  |  |  |  |
| SPEI in infancy: Humid sub-tropical |  |  | 1.050** | [1.018,1.083] |  |  |  |  |
| SPEI in infancy: Mountain |  |  | 0.833* | [0.716,0.969] |  |  |  |  |
|  |  |  |  |  |  |  |  |  |
| **SPEI latest monsoon season: Climate zone** |  |  |  |  |  |  |  |  |
| SPEI latest monsoon season: Tropical wet |  |  |  |  | 0.776 | [0.469,1.285] |  |  |
| SPEI latest monsoon season: Tropical wet & dry |  |  |  |  | 1.009 | [0.834,1.220] |  |  |
| SPEI latest monsoon season: Arid |  |  |  |  | 0.698 | [0.307,1.589] |  |  |
| SPEI latest monsoon season: Semi-arid |  |  |  |  | 1.064 | [0.871,1.300] |  |  |
| SPEI latest monsoon season: Humid sub-tropical |  |  |  |  | 1.102 | [0.969,1.253] |  |  |
| SPEI latest monsoon season: Mountain |  |  |  |  | 0.763 | [0.487,1.196] |  |  |
|  |  |  |  |  |  |  |  |  |
| **SPEI month of int.: Climate Zone** |  |  |  |  |  |  |  |  |
| SPEI month of int.: Tropical wet |  |  |  |  |  |  | 1.281 | [0.873,1.878] |
| SPEI month of int.: Tropical wet & dry |  |  |  |  |  |  | 1.02 | [0.922,1.129] |
| SPEI month of int.: Arid |  |  |  |  |  |  | 1.15 | [0.894,1.479] |
| SPEI month of int.: Semi-arid |  |  |  |  |  |  | 1.080+ | [0.987,1.181] |
| SPEI month of int.: Humid sub-tropical |  |  |  |  |  |  | 1.090* | [1.007,1.180] |
| SPEI month of int.: Mountain |  |  |  |  |  |  | 0.632* | [0.438,0.911] |
|  |  |  |  |  |  |  |  |  |
| **Climate zone** |  |  |  |  |  |  |  |  |
| Tropical wet & dry | 0.446*** | [0.357,0.557] | 0.949 | [0.790,1.140] | 0.702* | [0.506,0.973] | 0.390*** | [0.294,0.518] |
| Arid | 0.854 | [0.671,1.086] | 1.858*** | [1.539,2.243] | 0.643* | [0.430,0.963] | 3.040*** | [2.250,4.108] |
| Semi-arid | 1.907*** | [1.515,2.402] | 3.407*** | [2.825,4.109] | 0.367*** | [0.266,0.508] | 2.236*** | [1.692,2.954] |
| Humid sub-tropical | 0.576*** | [0.460,0.721] | 1.384*** | [1.149,1.666] | 0.214*** | [0.154,0.297] | 2.517*** | [1.886,3.359] |
| Mountain | 0.639*** | [0.570,0.717] | 1.181*** | [1.106,1.261] | 0.267*** | [0.141,0.506] | 14.788*** | [7.557,28.941] |
|  |  |  |  |  |  |  |  |  |
| Obs. | 110,319 |  | 188,712 |  | 188,529 |  | 225,577 |  |
| Pseudo R^2^ | 0.096 |  | 0.094 |  | 0.053 |  | 0.086 |  |

+<0.1, * <0.05, ** <0.01, *** <0.001. Notes: 95% CIs provided in parenthesis. All control variables as well as age splines, month of birth, year of birth, and district fixed effects are included but not displayed. Clustering at the district level. Climate zone classification based on Beck, H. E. *et al.,* Present and future Köppen-Geiger climate classification maps at 1-km resolution. *Sci. Data*. 5:180214 doi: 10.1038/sdata.2018.214 (2018).
