# Supplementary material for: Monsoon weather and early childhood health in India
Source: PLoS One. 2020 Apr 10;15(4):e0231479. doi: 10.1371/journal.pone.0231479 (PMC7147999; doi:10.1371/journal.pone.0231479)
Supplement: S3 Appendix — (DOCX) [file pone.0231479.s003.docx]

**S3 Appendix. Robustness analysis**

We perform several robustness analyses to check whether the results presented in the paper are sensitive to sample composition or model specification. Furthermore, we construct an alternative measure of climate shocks. The robustness analyses are performed for the main models on stunting, wasting and diarrhoea.

First, we test for sample composition effects by removing from the sample households who migrated between the time of exposure to climate shocks and the time of the interview. If households have moved long distance between the two periods, we would match them with the wrong climate data. The results of this analysis are presented in S10 Table. The effect of experiencing a SPEI shock in utero on stunting becomes slightly stronger compared to the baseline model. The effect of a SPEI shock during infancy on stunting are almost unchanged and the effect on a SPEI shock in the latest monsoon season prior to measurement on wasting remains insignificant.

Second, we add a control variable for low birth weight to the model. Low birth weight is generally associated with higher risk of undernutrition and is commonly used in models which explain stunting. However, we expect that birth weight would be affected by climate conditions during the in-utero period and therefore mediate the effect of climate shocks on child stunting. For this reason, we excluded it from the baseline model. The results of the extended model with low birth weight as a control variable are presented in S11 Table. We do not find substantial differences compared to the baseline model.

Finally, we construct an alternative measure of climate shocks, which is based on the monthly rainfall data from the CRU TS 3.25 database. We calculate a rainfall anomaly index, which measures deviations in monsoon season rainfall from the location-specific long-term mean. The following formula is used:

$${anomaly}_{j,t}=\frac{{level}_{j,t}-{mean}_{j}^{LT}}{{SD}_{j}^{LT}}$$

where ${level}_{j,t}$ is the total level of monsoon rainfall in location *j* during year *t*; ${mean}_{j}^{LT}$and ${SD}_{j}^{LT}$ are the long-term mean and standard deviation of monsoon season rainfall for the same location, where the long-term is the period from 1970 to 2016. Positive anomalies indicate excessive monsoon rainfall and negative anomalies indicate deficient monsoon rainfall. The results of the model with rainfall anomalies are presented in S12 Table below. The effects of rainfall anomalies on stunting are identical in direction to the effects of SPEI shocks, but smaller in size.

**S10 Table. Effects of monsoon season SPEI on undernutrition, children aged 0-5. Accounting for migration**

|  | stunted (HAZ<-2) | stunted (HAZ<-2) | wasted (WHZ<-2) |
| --- | --- | --- | --- |
|  | Odds Ratio | Odds Ratio | Odds Ratio |
| SPEI in utero | 1.058** |  |  |
|  | [1.022,1.095] |  |  |
| SPEI in infancy |  | 1.038** |  |
|  |  | [1.012,1.065] |  |
| SPEI latest monsoon season |  |  | 1.05 |
|  |  |  | [0.950,1.159] |
| Child is male | 1.136*** | 1.161*** | 1.170*** |
|  | [1.063,1.214] | [1.112,1.213] | [1.122,1.220] |
| Birth order | 1.083*** | 1.105*** | 1.014+ |
|  | [1.064,1.104] | [1.091,1.120] | [1.000,1.029] |
| Child is male: Birth order | 0.982+ | 0.971*** | 0.992 |
|  | [0.961,1.003] | [0.957,0.985] | [0.977,1.008] |
| Child is twin | 1.482*** | 1.446*** | 1.342*** |
|  | [1.255,1.751] | [1.287,1.625] | [1.207,1.492] |
| Mother's age | 0.983*** | 0.981*** | 0.998 |
|  | [0.978,0.987] | [0.978,0.984] | [0.994,1.001] |
| Mother's height | 0.941*** | 0.938*** | 0.991*** |
|  | [0.938,0.945] | [0.935,0.941] | [0.989,0.994] |
| Exposed to mass media | 0.961+ | 0.944*** | 0.987 |
|  | [0.923,1.001] | [0.915,0.974] | [0.953,1.022] |
| Primary education | 0.947* | 0.935*** | 0.924*** |
|  | [0.903,0.993] | [0.904,0.967] | [0.887,0.962] |
| Secondary education | 0.837*** | 0.817*** | 0.918*** |
|  | [0.801,0.875] | [0.791,0.844] | [0.885,0.952] |
| Higher education | 0.694*** | 0.660*** | 0.835*** |
|  | [0.636,0.757] | [0.622,0.700] | [0.781,0.893] |
| 2nd wealth quintile | 0.896*** | 0.871*** | 0.888*** |
|  | [0.856,0.939] | [0.842,0.901] | [0.851,0.926] |
| 3rd wealth quintile | 0.767*** | 0.747*** | 0.822*** |
|  | [0.724,0.814] | [0.714,0.781] | [0.781,0.866] |
| 4th wealth quintile | 0.618*** | 0.608*** | 0.774*** |
|  | [0.575,0.664] | [0.576,0.642] | [0.729,0.822] |
| Top wealth quintile | 0.537*** | 0.509*** | 0.707*** |
|  | [0.491,0.586] | [0.474,0.546] | [0.657,0.762] |
| Sanitation facility is improved | 0.962+ | 0.948** | 0.964* |
|  | [0.921,1.005] | [0.918,0.979] | [0.932,0.998] |
| Household head is female | 1.026 | 1.02 | 0.977 |
|  | [0.979,1.075] | [0.985,1.057] | [0.940,1.016] |
| Number of under-5 children | 1.060*** | 1.068*** | 0.951*** |
|  | [1.039,1.081] | [1.052,1.085] | [0.936,0.967] |
| Scheduled caste | 1.208*** | 1.245*** | 1.069* |
|  | [1.132,1.288] | [1.191,1.301] | [1.016,1.124] |
| Scheduled tribe | 1.195*** | 1.190*** | 1.149*** |
|  | [1.109,1.287] | [1.126,1.257] | [1.078,1.226] |
| Other backward caste | 1.096*** | 1.128*** | 1.054* |
|  | [1.043,1.151] | [1.086,1.171] | [1.009,1.101] |
| Muslim religion | 1.096** | 1.139*** | 1.04 |
|  | [1.032,1.164] | [1.090,1.191] | [0.989,1.094] |
| Christian religion | 0.956 | 0.961 | 1.039 |
|  | [0.834,1.094] | [0.881,1.048] | [0.921,1.172] |
| Other religion | 0.915 | 0.929+ | 1.043 |
|  | [0.812,1.030] | [0.858,1.005] | [0.948,1.149] |
| Obs. | 79,562 | 162,203 | 178,621 |
| Pseudo R^2^ | 0.097 | 0.092 | 0.053 |

+<0.1, * <0.05, ** <0.01, *** <0.001. Notes: 95% CIs provided in parenthesis. Age splines, month of birth, year of birth, and district fixed effects are included but not displayed. Clustering at the district level.

**S11 Table. Effects of monsoon season SPEI on undernutrition, children age 0-5. Controlling for birth weight**

|  | stunted (HAZ<-2) | stunted (HAZ<-2) | wasted (WHZ<-2) | diarrhoea |
| --- | --- | --- | --- | --- |
|  | Odds Ratio | Odds Ratio | Odds Ratio | Odds Ratio |
| SPEI in utero | 1.049** |  |  |  |
|  | [1.017,1.081] |  |  |  |
| SPEI in infancy |  | 1.037** |  |  |
|  |  | [1.011,1.064] |  |  |
| SPEI latest monsoon season |  |  | 1.043 |  |
|  |  |  | [0.949,1.146] |  |
| SPEI month of interview |  |  |  | 1.065* |
|  |  |  |  | [1.009,1.124] |
| Child is male | 1.151*** | 1.152*** | 1.160*** | 1.117*** |
|  | [1.095,1.210] | [1.110,1.196] | [1.113,1.210] | [1.061,1.175] |
| Birth order | 1.082*** | 1.097*** | 1.011 | 1.041*** |
|  | [1.064,1.100] | [1.084,1.111] | [0.996,1.025] | [1.022,1.061] |
| Child is male: Birth order | 0.980* | 0.975*** | 0.996 | 0.987 |
|  | [0.962,0.998] | [0.962,0.988] | [0.980,1.013] | [0.969,1.005] |
| Child is twin | 1.395*** | 1.358*** | 1.234*** | 0.847* |
|  | [1.214,1.603] | [1.215,1.519] | [1.108,1.374] | [0.723,0.991] |
| Low birth weight | 1.348*** | 1.309*** | 1.292*** | 1.278*** |
|  | [1.288,1.410] | [1.265,1.355] | [1.243,1.342] | [1.213,1.347] |
| Mother's age | 0.982*** | 0.981*** | 0.998 | 0.987*** |
|  | [0.978,0.986] | [0.978,0.984] | [0.995,1.002] | [0.982,0.992] |
| Mother's height | 0.939*** | 0.937*** | 0.992*** | 1.002+ |
|  | [0.936,0.942] | [0.935,0.940] | [0.989,0.994] | [1.000,1.005] |
| Exposed to mass media | 0.965* | 0.945*** | 0.993 | 1.015 |
|  | [0.932,0.999] | [0.918,0.973] | [0.959,1.029] | [0.970,1.062] |
| Primary education | 0.945* | 0.935*** | 0.919*** | 1.171*** |
|  | [0.904,0.987] | [0.906,0.964] | [0.883,0.956] | [1.111,1.235] |
| Secondary education | 0.838*** | 0.817*** | 0.915*** | 1.109*** |
|  | [0.806,0.873] | [0.793,0.842] | [0.883,0.949] | [1.054,1.167] |
| Higher education | 0.677*** | 0.664*** | 0.841*** | 1.005 |
|  | [0.633,0.723] | [0.630,0.701] | [0.789,0.897] | [0.928,1.088] |
| 2nd wealth quintile | 0.888*** | 0.870*** | 0.886*** | 0.977 |
|  | [0.851,0.927] | [0.841,0.899] | [0.849,0.924] | [0.927,1.030] |
| 3rd wealth quintile | 0.763*** | 0.744*** | 0.825*** | 0.959 |
|  | [0.723,0.805] | [0.713,0.775] | [0.784,0.869] | [0.898,1.024] |
| 4th wealth quintile | 0.619*** | 0.604*** | 0.772*** | 0.943 |
|  | [0.582,0.660] | [0.574,0.635] | [0.727,0.819] | [0.872,1.021] |
| Top wealth quintile | 0.524*** | 0.498*** | 0.711*** | 0.888** |
|  | [0.484,0.567] | [0.466,0.531] | [0.661,0.765] | [0.814,0.968] |
| Sanitation facility is improved | 0.956* | 0.949*** | 0.969+ | 0.933** |
|  | [0.920,0.994] | [0.921,0.977] | [0.937,1.003] | [0.892,0.976] |
| Household head is female | 1.028 | 1.017 | 0.978 | 1.009 |
|  | [0.986,1.072] | [0.984,1.051] | [0.941,1.017] | [0.961,1.060] |
| Number of under-5 children | 1.052*** | 1.069*** | 0.951*** | 0.887*** |
|  | [1.034,1.071] | [1.054,1.085] | [0.936,0.967] | [0.868,0.906] |
| Scheduled caste | 1.187*** | 1.232*** | 1.074** | 1.029 |
|  | [1.124,1.254] | [1.181,1.285] | [1.020,1.130] | [0.970,1.091] |
| Scheduled tribe | 1.163*** | 1.174*** | 1.169*** | 0.924+ |
|  | [1.092,1.239] | [1.115,1.237] | [1.097,1.245] | [0.852,1.001] |
| Other backward caste | 1.090*** | 1.116*** | 1.054* | 1.008 |
|  | [1.044,1.139] | [1.076,1.156] | [1.009,1.100] | [0.956,1.062] |
| Muslim religion | 1.099*** | 1.144*** | 1.048+ | 1.148*** |
|  | [1.043,1.159] | [1.098,1.193] | [0.999,1.100] | [1.084,1.216] |
| Christian religion | 1.005 | 0.972 | 1.03 | 1.011 |
|  | [0.899,1.122] | [0.898,1.051] | [0.919,1.154] | [0.854,1.197] |
| Other religion | 0.944 | 0.928+ | 1.041 | 1.167* |
|  | [0.862,1.034] | [0.860,1.002] | [0.949,1.143] | [1.006,1.353] |
| Obs. | 107,910 | 184,419 | 184,186 | 220,557 |
| Pseudo R^2^ | 0.098 | 0.095 | 0.054 | 0.086 |

+<0.1, * <0.05, ** <0.01, *** <0.001. Notes: 95% CIs provided in parenthesis. Age splines, month of birth, year of birth, and district fixed effects are included but not displayed. Clustering at the district level.

**S12 Table. Effects of monsoon season climate on undernutrition, children aged 0-5. Precipitation anomalies**

|  | stunted (HAZ<-2) | stunted (HAZ<-2) | wasted (WHZ<-2) |
| --- | --- | --- | --- |
|  | Odds Ratio | Odds Ratio | Odds Ratio |
| Pre anom in utero | 1.024** |  |  |
|  | [1.007,1.041] |  |  |
| Pre anom in infancy |  | 1.020** |  |
|  |  | [1.006,1.034] |  |
| Pre anom latest monsoon season |  |  | 1.005 |
|  |  |  | [0.951,1.062] |
| Child is male | 1.143*** | 1.151*** | 1.155*** |
|  | [1.087,1.201] | [1.108,1.195] | [1.108,1.204] |
| Birth order | 1.080*** | 1.098*** | 1.012 |
|  | [1.062,1.097] | [1.085,1.111] | [0.997,1.026] |
| Child is male: Birth order | 0.982* | 0.974*** | 0.996 |
|  | [0.964,1.000] | [0.961,0.987] | [0.981,1.012] |
| Child is twin | 1.475*** | 1.430*** | 1.298*** |
|  | [1.285,1.692] | [1.280,1.597] | [1.168,1.443] |
| Mother's age | 0.981*** | 0.981*** | 0.998 |
|  | [0.978,0.985] | [0.978,0.983] | [0.994,1.001] |
| Mother's height | 0.939*** | 0.937*** | 0.991*** |
|  | [0.936,0.942] | [0.934,0.940] | [0.989,0.994] |
| Exposed to mass media | 0.969+ | 0.946*** | 0.99 |
|  | [0.936,1.003] | [0.919,0.974] | [0.956,1.024] |
| Primary education | 0.949* | 0.934*** | 0.922*** |
|  | [0.909,0.991] | [0.905,0.964] | [0.887,0.959] |
| Secondary education | 0.840*** | 0.817*** | 0.915*** |
|  | [0.807,0.873] | [0.792,0.842] | [0.882,0.948] |
| Higher education | 0.673*** | 0.661*** | 0.836*** |
|  | [0.630,0.719] | [0.627,0.697] | [0.784,0.891] |
| 2nd wealth quintile | 0.883*** | 0.870*** | 0.888*** |
|  | [0.846,0.920] | [0.842,0.899] | [0.852,0.925] |
| 3rd wealth quintile | 0.756*** | 0.742*** | 0.826*** |
|  | [0.717,0.797] | [0.712,0.773] | [0.785,0.869] |
| 4th wealth quintile | 0.615*** | 0.604*** | 0.772*** |
|  | [0.578,0.655] | [0.574,0.636] | [0.728,0.819] |
| Top wealth quintile | 0.518*** | 0.497*** | 0.712*** |
|  | [0.479,0.561] | [0.465,0.530] | [0.662,0.765] |
| Sanitation facility is improved | 0.957* | 0.946*** | 0.967* |
|  | [0.921,0.995] | [0.918,0.974] | [0.935,1.000] |
| Household head is female | 1.027 | 1.013 | 0.976 |
|  | [0.985,1.070] | [0.981,1.047] | [0.939,1.013] |
| Number of under-5 children | 1.050*** | 1.068*** | 0.951*** |
|  | [1.032,1.069] | [1.053,1.083] | [0.935,0.967] |
| Scheduled caste | 1.189*** | 1.233*** | 1.071** |
|  | [1.125,1.256] | [1.183,1.285] | [1.018,1.125] |
| Scheduled tribe | 1.165*** | 1.173*** | 1.166*** |
|  | [1.093,1.241] | [1.114,1.236] | [1.095,1.242] |
| Other backward caste | 1.091*** | 1.115*** | 1.052* |
|  | [1.045,1.140] | [1.077,1.156] | [1.008,1.098] |
| Muslim religion | 1.102*** | 1.147*** | 1.050* |
|  | [1.046,1.162] | [1.100,1.196] | [1.001,1.102] |
| Christian religion | 0.993 | 0.966 | 1.026 |
|  | [0.890,1.108] | [0.893,1.045] | [0.914,1.151] |
| Other religion | 0.954 | 0.935+ | 1.026 |
|  | [0.871,1.044] | [0.868,1.008] | [0.936,1.124] |
| Obs. | 110,319 | 188,712 | 188,529 |
| Pseudo R^2^ | 0.096 | 0.094 | 0.053 |

+<0.1, * <0.05, ** <0.01, *** <0.001. Notes: 95% CIs provided in parenthesis. Age splines, month of birth, year of birth, and district fixed effects are included but not displayed. Clustering at the district level.
