# Supplementary material for: Monsoon weather and early childhood health in India
Source: PLoS One. 2020 Apr 10;15(4):e0231479. doi: 10.1371/journal.pone.0231479 (PMC7147999; doi:10.1371/journal.pone.0231479)
Supplement: Attachment — Submitted filename: Response to reviewers.docx [file pone.0231479.s005.docx]

**COMMENTS BY REVIEWER 2**

1. Minor things:
   209-210: For the benefit of researchers without a geophysical background, clarify why it is important that SPEI includes temperature: because temp influences evapotranspiration. Thus, in very warm, sunny conditions, excessive rainfall will quickly dry up, whereas in cooler, cloudy conditions, excessive water will stick around longer, potentially further affecting sanitary conditions. SPEI accounts for this in a way that rainfall alone does not.

**Response:** Thank you for this suggestion. We have included the following text on lines 280-284:

“It is important to account for the effect of temperature on evapotranspiration in addition to rainfall. For example, in sunny places with high temperatures, excessive rainfall will quickly dry up, whereas in cooler and cloudier places, excessive water will accumulate and remain for longer, potentially affecting sanitary conditions. SPEI accounts for this in a way that rainfall alone does not.”

2. 446: This is confusing. Do you mean "a few studies"?

**Response:** Yes, thank you for the correction.

3. Finally, I would love to see some publicly available code for this analysis, perhaps as a github repository. This would be useful for future researchers interested in building on your results.

**Response:** We are open to share the codes for producing this analysis on github and other platforms to facilitate future research on this topic.
